# Vulnerability in nursing science: a principle-based concept analysis

**DOI:** 10.1186/s12912-026-05523-w

**Published:** 2026-10-16

**Authors:** Miriam Läpple, Martina Hasseler

**Affiliations:** 1 Department of Nursing Science, Faculty of Educational Sciences, University of Koblenz, Universitätsstraße 1, Koblenz, 56070 Germany; 2 Faculty of Health Sciences, Ostfalia University of Applied Sciences, Wolfsburg, Germany

**Keywords:** Concept analysis, Health disparate minority and vulnerable populations, Health policy, Needs assessment, Social determinants of health, Social vulnerability

## Abstract

**Background:**

The term “vulnerability” is widely used across disciplines and variably defined within nursing, reflecting differing theoretical and methodological assumptions. This ambiguity hinders coherent application in research and practice. A broad understanding of vulnerability in healthcare is susceptibility to risks and/or illnesses, implying the need for special protection or treatment.

**Methods:**

To clarify the concept of vulnerability in nursing, its disciplinary meanings were synthesized and its maturity was evaluated by using a principle-based concept analysis according to Penrod and Hupcey (2005), which was conducted on nursing literature. Databases MEDLINE via PubMed, CINAHL and LIVIVO were systematically searched with defined keywords, and citation searching. Of 1,169 records identified, 49 studies (1983–2024) were included. The evidence was evaluated using the principles of principle-based concept analysis: epistemological adequacy, pragmatic utility, linguistic clarity, and logical coherence.

**Results:**

Three diverging perspectives were identified: (1) vulnerability as a universal human condition; (2) vulnerability as an individual, risk-oriented notion; and (3) vulnerability as a multifactorial state influenced by social determinants of health. Each perspective shapes nursing’s approach to individuals in its own way, focusing on different methodologies. The three perspectives have been combined into an integrative concept for nursing. It addresses the individual, community, and healthcare policy levels. The multidimensionality of vulnerability is suitable to support the notion of nursing care for the individual with a view to the social determinants of health. An assessment of the concept across the principles indicates that it is partially mature and requires further theoretical and empirical development.

**Conclusions:**

The analysis provides an integrative concept of vulnerability for nursing science which can serve as a foundation for its disciplinary use and thematic relevance. Further work should develop discriminant definitions vis-à-vis related constructs, advance valid operational measures, and test the concept empirically across settings to support assessment, intervention design, and system-level health policy, health-promotion, and disease prevention strategies.

**Trial registration:**

Not applicable.

**Supplementary Information:**

The online version contains supplementary material available at https://doi.org/10.1186/s12912-026-05523-w.

## Background

### Vulnerability in health care: definitions and practice

Etymologically, vulnerability derives from the Latin “vulnus” (“wound”), connoting susceptibility to harm [1]. In mainstream healthcare discourse, the term “vulnerability” is often used to describe a categorization of designated vulnerable groups”,^1^ including people living with dementia, older adults, children, or persons with disabilities. Group-based vulnerability is operationalized through targeted protective measures or differentiated healthcare to mitigate condition-specific hazards, such as the increased threat of infections for older people as witnessed in the pandemic or during pregnancy for women [2], or violence against children or people with disabilities who are not (yet) able to defend themselves adequately [3, 4]. In practice, social or expert attributions, (e.g., by epidemiologists in the pandemic [5] or politicians responsible for creating structural health frameworks [6]) constitute and influence the discourse of who is categorized as “vulnerable” and which actions would be justified to protect them. Corresponding measures include ethical reasoning, structured assessment and planning of care pathways, and organizational arrangements that shape interaction with—and participation of—so-called vulnerable persons (e.g., [7–9].

### The gap between categorial attribution and the professional approach of nursing

While categorial attribution dominates health care, there is limited, discipline-specific guidance for nurses to specify individual-level goals and actionable interventions grounded in a theoretical understanding on vulnerability. However, the new definition of the International Council of Nurses explicitly states that nurses must “[…] provide people-centred, compassionate clinical and social care, […] advance public and population health, and foster safe and sustainable environments.” [10, p. 12].

According to this definition, nurses are called to focus more than ever on the social determinants of health and health equity. In primary healthcare, for instance, nurses pursue professional goals such as leading, educating, researching, advocating, and shaping policy to improve health outcomes [11]. To do so, nurses need to detect and work with relevant health care concepts. Vulnerability is such a concept, as it is linked with individually and structurally shaped social determinants of health that influence access to health care. In order to plan, justify, carry out, and evaluate individual-level goals and actionable interventions for healthcare provision that acknowledges social determinants of health, nursing needs a framework that recognizes and works with vulnerability from a theoretical foundation.

Currently, the categorial notion of vulnerability with regard to so-called vulnerable groups dominates nursing science [12]. In contrast, there are also philosophical analyses that understand vulnerability as a fundamental anthropological condition that exceeds the categorial attributions [13, 14]. This understanding asserts that human beings are inherently vulnerable because we are interconnected with our environment and community and dependent on relationships. Sudden natural hazards or episodes of violence encountered “in the wrong place at the wrong time” illustrate this fundamental fragility and precarity of life [13].

Heaslip [15] states that references to this conception of human vulnerability exist in nursing science, but they are vague and influence nursing discourse to an unknown degree. If vulnerability is inherent and relational in this sense, important implications follow for nursing: Specifically, a theoretically based, relational understanding is required that transcends the categorial attribution of vulnerable versus non-vulnerable people. Without this understanding, nursing runs the risk of merely reproducing this categorization and failing to focus on the causes and relations concerning the social determinants of health and health inequity. Thus, this article aims to review existing concepts and understandings of vulnerability in nursing science and propose a literature- and theory-based definition of vulnerability. This article addresses the following research questions:

1. Which concepts or understandings of vulnerability are used in nursing science?
2. How can these concepts be synthesized into an integrative definition of vulnerability in nursing science?

## Methods

As the objective of this article is to clarify vulnerability in nursing science, this concept analysis used a principle-based, literature-based approach, following the methodological framework of Penrod and Hupcey [16], originally proposed by Morse and colleagues [17, 18].

Specifically, this framework is suitable for critically analysing differing notions of vulnerability possibly found in the literature. As Morse et al. [19] state, it “(…) refers to a process of inquiry that explores concepts for their level of development or maturity as revealed by their internal structure, use, representativeness, and/or relations to other concepts.” The principles represent systematically differing dimensions of the concept and thus provide perspectives for further development within four criteria: epistemology, pragmatics, linguistics, and logic. The procedure of conducting a concept analysis [16] involves the following steps: (1) structured retrieval of literature, (2) screening and selection of eligible publications, (3) analysis of the included sources, and (4) evaluation of the concept based on the four criteria.

### Literature review

Penrod and Hupcey [16] state that the structured retrieval of literature should be adequate and appropriate. To adhere to these recommendations, we conducted a systematic literature search in accordance with the PRISMA-ScR guideline. The guideline was chosen since there is not yet a specific reporting guideline for concept analyses, and because it addresses to provide an overview of evidence and research gaps whereas systematic reviews aim to answer concretly defined (clinical) research questions [20].^2^ However, as the manuscript primarily concerns the principle-based concept analysis method, this subsection is not structured as proposed by the guideline, but provides all the necessary criteria for transparency within the literature search.

The search was designed as specific and a priori, and was conducted in CINAHL, MEDLINE (via PubMed), and LIVIVO, a German life sciences index that includes scientific and gray literature. Where appropriate, we used controlled vocabulary and validated keywords: These were iteratively tested for validity and adapted to each database’s thesaurus; as presented in Table 1.

**Table 1:** Search Terms and Boolean Operators

	CINAHL	MEDLINE	LIVIVO
1	All text "Vulnerability"	Vulnerability"[all fields]	Open Search="Vulnerability"
2	Major subject “Nursing Practice”	“Nursing“[MeSH]	Keyword="Nursing”
3	All text “Concept” OR all text “phenomenon” OR all text “understanding”	“Concept“[all fields] OR “phenomenon“[all fields] OR “understanding“[all fields]	Open Search “Concept” OR Open Search="phenomenon” OR Open Search="understanding”
4	#1 AND #2 AND #3	#1 AND #2 AND #3	#1 AND #2 AND #3

We applied the Major Subject “Nursing Practice” in CINAHL and the MeSH term “Nursing” in MEDLINE. However, regarding “vulnerability,” we decided against using subject headings in the primary search: In CINAHL, the major heading for vulnerability reflects susceptibility, and in MEDLINE, the MeSH descriptor is “Vulnerable Populations.” Since our analysis aims to capture the broader concept of vulnerability beyond susceptibility or group-based attributes, these controlled terms were deemed too restrictive for our purposes. Consequently, we searched “vulnerability” as an “all text” term without applying truncation. This high-specificity approach was chosen to capture papers explicitly dealing with the conceptualization itself, combined with methodological terms (“concept,” “phenomenon,” “understanding”). Despite not explicitly searching for synonyms like “risk” or “susceptibility” in the search string, the literature review revealed a frequent conflation of vulnerability with these terms within the retrieved texts, indicating a linguistic ambiguity of the concept.

**Table 2:** Inclusion and exclusion criteria

	Inclusion	Exclusion
Language	No restriction	
Publication year	No restriction	
Population	Vulnerability of human beings (people, patients, clients) in the context of nursing care	Vulnerability of nurses, health systems, technology, management, environment, or education
Context	Explanation or definition of vulnerability is stated or exemplified	No stated explanation or definition of vulnerability; empirical reports that labeled findings (e.g., categories) as “vulnerability” without articulating a conceptual or definitiory framework
Publication type	Journal articles, Research Book, Contributions in Textbooks, Gray Literature	Conference Papers, Editorials, Commentaries, Unpublished Contributions

Table 2 provides an overview of inclusion and exclusion criteria. Also, Supplemental Material 1 provides additional information on the systematics of the literature search. Also, please refer to the adjusted PRISMA-ScR Checklist.

Non-English records were translated using institutionally supported, AI-assisted translation software (DeepL). To address possible gaps in the initial retrieval caused by the specific search strategy, we conducted a single iteration of backward citation searching using citationchaser [22], in line with recommendations to strengthen methodological rigor [23]. The reference lists of the included studies from the primary database search served as the seed sets. All newly identified records were screened using the same eligibility criteria. Deduplication combined manual checks with Rayyan [24]. Reporting for both the primary and backward searches followed the PRISMA Flow Chart to ensure transparency [21].

We conducted data extraction using MAXQDA 24 [25] with a predefined template that captured bibliographic and methodological descriptors (please see Supplemental Material 2). This generated a descriptive overview of the data. For the analytic appraisal, the study texts were coded deductively against four guiding principles: epistemological adequacy, pragmatic utility, linguistic clarity, and logical coherence. Principle-relevant segments were organized into data extraction tables, and differentiation criteria were applied to compare and evaluate the concept across studies.

### The principle-based concept analysis

To clarify the concept of vulnerability and its relevance to nursing, we used a principle-based concept analysis. Unlike concept analysis approaches that incorporate colloquial, artistic, or media sources (e.g., [26, 27],^3^ the principle-based method privileges empirical and theoretical publications and resources, aiming to synthesize the best available scientific knowledge into a working definition and attribute structure. Since scientific knowledge evolves, the resulting definition is provisional and requires periodic reassessment with respect to content, identified gaps, and (inter-)disciplinary scope. The epistemological goal is not absolute truth about vulnerability in nursing but rather the most defensible concept analysis supported by the scientific literature.

**Fig. 1:**
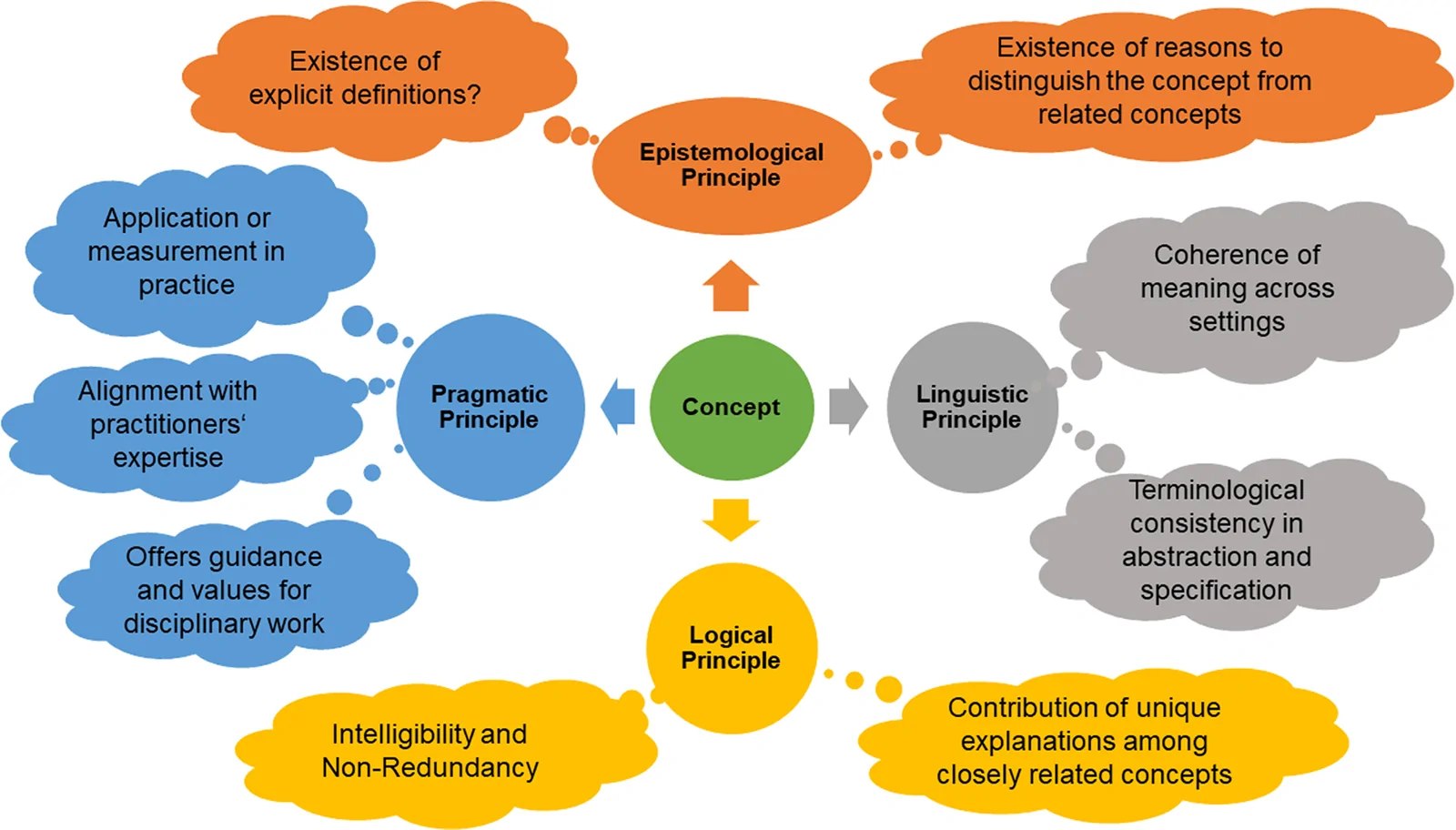
The principles’ perspectives

Analytically, the method appraises the concept across four principles: epistemological adequacy, pragmatic utility, linguistic clarity, and logical coherence. This analytical approach provides a transparent assessment of the state of knowledge at different levels of abstraction [16, 29]. Figure 1 provides a concise overview of these principles and their specific perspectives.

Concept maturity reflects the degree of definitional clarity, theoretical coherence, and empirical applicability. Mature concepts are precisely defined, theoretically consistent, and operationalizable. They exhibit stable relationships and clear boundaries that minimize overlap [18]. By contrast, immature concepts remain ambiguous, are used inconsistently, and are resistant to measurement. Therefore, the method of principle-based concept analysis is well suited for advancing theoretical, conceptual, and empirical research on vulnerability in nursing science: By applying these principles, we can derive concrete implications for the applicability of vulnerability in nursing and formulate targeted research questions.

## Results

**Fig. 2:**
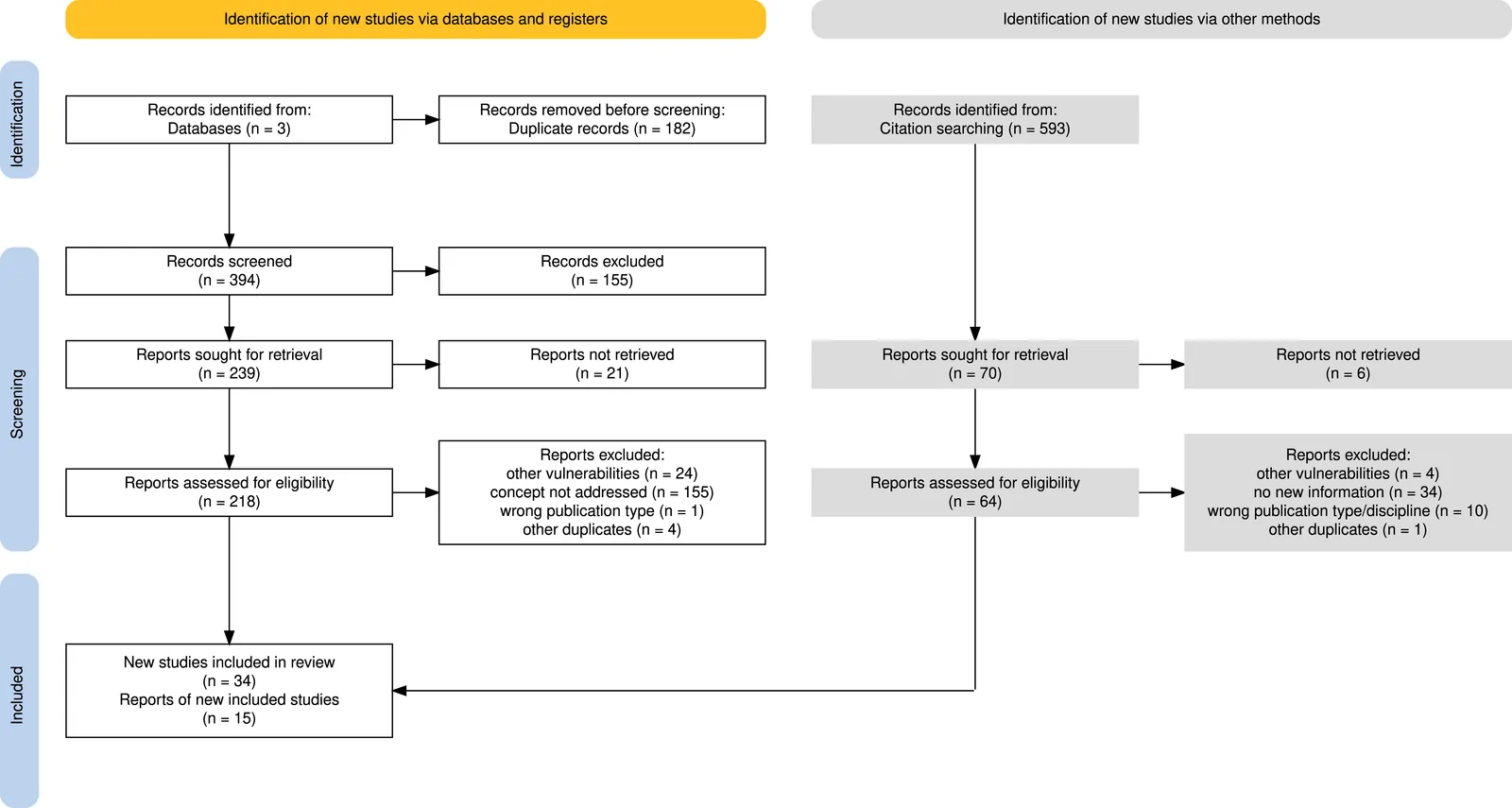
PRISMA Flow Chart

The search for the concept yielded 1,169 records. Two reviewers independently screened 987 titles and abstracts, assessed 282 full texts, and ultimately included 49 studies: 48 journal articles and 1 edited volume, as shown in Fig. 2 [30].

All included records originate from nursing and were published between 1983 and 2024. Studies were conducted in Brazil (*n* = 13), Norway (*n* = 2), Finland (*n* = 1), Sweden (*n* = 2), Denmark (*n* = 1), the United States (*n* = 11), Canada (*n* = 4), Great Britain (*n* = 12), Australia (*n* = 2), and Austria (*n* = 1). Sixteen were empirical investigations and 33 were theoretical or methodological contributions. ML extracted the data and MH verified its plausibility and accuracy. Any disagreements were resolved through discussion and consensus. The following section presents the results first in terms of content, then classified according to the four principles and their implications for nursing. Three framing perspectives on vulnerability are derived across the included studies, each carrying distinct implications for epistemological, pragmatic, linguistic, and logical criteria. These contextual differences manifest as divergent operational definitions and research methodologies.

### Epistemological views: identified perspectives of vulnerability

1. First, vulnerability is conceptualized as a universal human condition arising from our inherent exposure to potential harm [31–33]. Authors underscore human fragility and mortality [31, 34], existential uncertainty [9, 33] and relational dependence, which makes vulnerability mutual [34, 35]. Described as ordinary [33] or "emic" vulnerability [36] linked to lived experience, this perspective varies by self-perception of challenges and harm [37]. It permits growth [34], characterized by stress, anxiety, or threats to autonomy [37, 38], yet openness to experience can be positive [32]. This perspective is rooted in anthropological and philosophical approaches [14].
2. Second, vulnerability is conceptualized as impaired self-protection or an increased likelihood of harm, emphasizing individual risk factors [9]. Associated with susceptibility or frailty [31, 32] and Sellman’s “more-than-ordinary” vulnerability [33], this “etic” perspective [36] anchors in group-level demographics or situational and adverse exposures. Designated populations include children, older adults, those with mental health issues or disabilities, and socioeconomically disadvantaged groups. Nichiata et al. [12] summarize this as addressing alterations from biological or psychological “normalcy.” Individuals are deemed vulnerable based on their constitution [39], often within a biomedical context.
3. Third, building on the first two, vulnerability is viewed as a multifactorial characteristic, influenced by dimensions beyond individual properties [12]. Authors describe it as the capacity to decide or act within risky situations, determined by various individual, familial, cultural, social, political, biological, and economic factors [40], including epidemiological and psychological aspects [41]. Interrelations are identified as individual (resources for safe behaviors), social (access to care, information, rights), and programmatic^4^ or institutional axes (prevention, promotion, care) [6, 37, 42–44]. Exposure to harm and coping capacity depend heavily on these factors; individuals are not deemed responsible when structural factors limit self-care opportunities [45, 46]. Vulnerability thus serves as an indicator of social inequality and health inequity [12], defined by rights entitlement, participation, and economic policies [40]. Epistemologically, the included studies approach vulnerability differently within specific nursing fields; for example, acute care yields a different understanding than community-based or long-term care.

### The pragmatic principle

According to Penrod and Hupcey [16, 29], the pragmatic principle evaluates a concept’s usefulness within the discipline and its degree of operationalization. Usefulness implies that the concept resonates with practitioners’ experiences and guides disciplinary work: Operationalization, manifested through specific instruments, measurements, or assessments, is considered a high level of pragmatic development. The analysis reveals how these two aspects unfold across the three perspectives:

1. Vulnerability as a basic human condition focuses on the people’s experiences of vulnerability and their own assessment of their ability to cope with challenges and crises [33]. This perspective uses qualitative and phenomenological research methods to capture how individuals experience, evaluate, and perceive potentially vulnerable events, highlighting the differences in how these are perceived as challenging. Spiers [36] suggests that people do not experience vulnerability if they maintain the capability to manage challenges. This experience is significantly impacted by sensitivity to one’s own emotions and those of others, which often grows stronger with age [32]. Angel and Vatne [35] describe experiences such as verbal or physical attacks, viruses, workloads, and accidents in relation to individuals’ changing physical and mental resources. Carel [47] proposes a phenomenological approach to experiences of vulnerability, emphasizing the lived body and embodiment. Schrems [9] refers to Kipnis’s [48] model, which considers discrete patterns of cognitive, juridical, deferential, medical, allocational, and infrastructural vulnerability and notes that these patterns can be used to assess an individual’s vulnerability in an ongoing, participatory process, particularly within research ethics.
2. This perspective focuses on operationalizing vulnerability through quantitative measures to assess the degree of vulnerability and risk concerning adverse outcomes, notably increased morbidity and mortality [49]. Key parameters contributing to this understanding of vulnerability include frailty, weakness, chronic illness, and declines in functional capacities [32]. Relevant risk factors are multifaceted and include age, gender, socioeconomic status, race, level of education, emotional development, knowledge, and personal beliefs [12, 50]. Additional factors influencing this perspective include substance use, sexual practices, religious beliefs, potential for mental health conditions, and early pregnancy [51]. These factors are rooted in public health and substantiate assessments of individual or group vulnerability. Various scales and assessment tools are used to evaluate the vulnerability of older adults through self-reporting or proxy assessments [39, 52].
3. The third perspective incorporates the social determinants of health and addresses the axes of individual, social, and programmatic or institutional factors. Individual factors encompass health literacy [43, 44] and the individual’s capacity to evaluate and modify lifestyle habits [41, 51, 53]. Williams [54] highlights the significance of self-esteem, coping skills, and parental role models as crucial elements for vulnerable families. Social or socioeconomic influences include freedoms related to thought and expression [41], cultural and gender considerations [12, 51], as well as structural determinants like economic instability contributing to unemployment, adverse living conditions, and issues related to financial stability. These elements manifest in access to goods, purchasing power, nutritional adequacy, and access to education and healthcare [6, 43, 51]. Furthermore, combinations of stressors such as spousal loss, decreased income, and diminished social networks are acknowledged [32]. Institutional and programmatic factors include the provision of stable and continuous funding for welfare services and human rights initiatives [12, 55], advancements in addressing social determinants of health, and the development of connections between institutions, programs, and affected populations [41, 51].

According to the pragmatic principle, there are three distinct approaches to operationalizing vulnerability within the included studies: (1) through phenomenological assessment of lived experience, (2) via quantitative risk measurements, and (3) by evaluating structural and social determinants of health.

### The linguistic principle

The term “vulnerability” has dual linguistic characteristics: The concept of vulnerability as a human condition carries a positive connotation. It is associated with potential for growth and resilience [13, 47]. Conversely, the term often denotes heightened risk and the likelihood of negative outcomes, particularly within the biomedical framework [56]. The lack of consensus on the term’s definition complicates its application, as vulnerability can refer broadly to individuals at risk across various contexts [33]: Schrems [9] advocates for a relational and context-sensitive research approach when working with populations identified as vulnerable. On the other hand, individual vulnerability is understood in the context of broader social, programmatic, and institutional structures that generate vulnerability [12, 39]. In summary, vulnerability is linguistically situated as both a monocausal-individual and a relational, context-sensitive term. This highlights the complexity of its interpretation and application across research and practice.

### The logical principle

In the biomedical context, the terms “risk” and “vulnerability” are frequently used interchangeably. However, they possess distinct connotations. Risk is primarily associated with individual susceptibility to adverse health outcomes, whereas vulnerability encompasses a broader spectrum that includes quality of life. Some researchers have identified a lack of conceptual clarity surrounding vulnerability [31, 38]. Others highlight the relevance of temporal and logical causalities in understanding these concepts [12, 33, 50].

Vulnerability includes individual risk factors that heighten disease susceptibility [36], and it is also informed by a confluence of personal and environmental factors [38, 50]. Some research suggests that these terms are often used interchangeably, with frailty characterized as a state [32, 39, 57] or precursor [58] of vulnerability. Furthermore, concepts such as marginalization, stigma, and oppression exhibit a logical interrelationship with vulnerability: The designation of individuals as vulnerable often signifies a level of powerlessness, potentially leading to stigmatization and marginalization [9, 15]. Oppression is understood to occur at personal, cultural, and structural levels, perpetuating vulnerability through power imbalances [46, 59]. Conversely, resilience serves as an opposing construct to vulnerability. It emphasizes coping strategies and the availability of individual, environmental, or structural resources in the face of life crises or illnesses [35, 38]. Dorsen [56] states that vulnerability can be viewed as an opportunity to enhance individual or community resilience. Thus, the analysis reveals that vulnerability is logically related to various surrounding concepts, such as risk, frailty, susceptibility, and resilience, yet lacks clear delimitation in the current nursing discourse.

### Relations of vulnerability: antecedents, attributes, and consequences

**Fig. 3:**
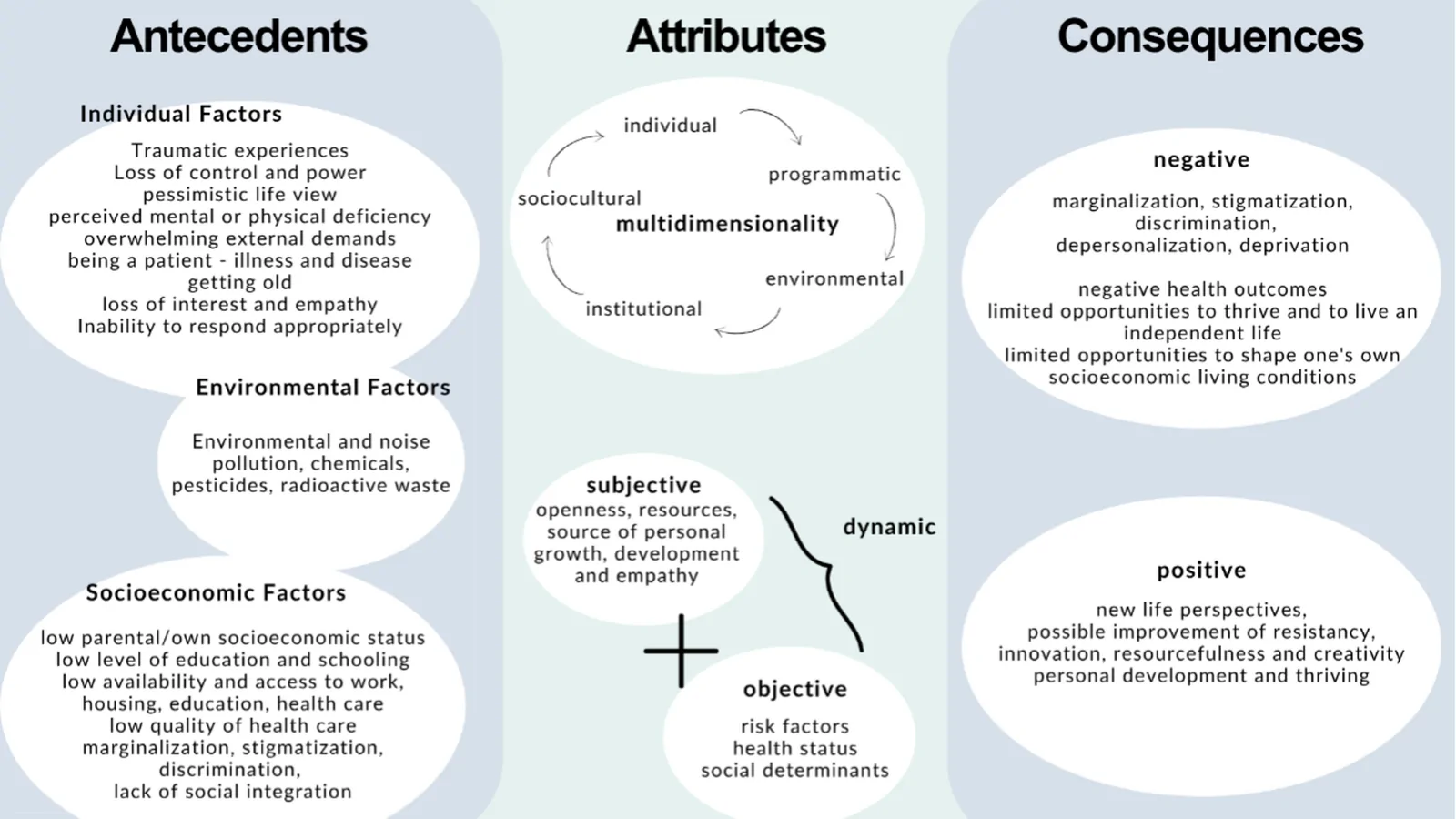
Antecedents, Attributes, and Consequences of Vulnerability in Nursing Science

The following section provides a summary of the results, presenting the concept, its antecedents, attributes, and consequences in Fig. 3, based on the literature reviewed.

The antecedents, attributes, and consequences of vulnerability are interdependent and mutually influential, shaped by a variety of factors reflected in the multidimensional nature of vulnerability’s attributes, encompassing individual, programmatic, environmental, institutional, and sociocultural dimensions. Vulnerability encompasses a dynamic relationship of both subjective and objective factors integrated within the conceptual framework [49, 60]. Objective factors are described by quantifying vulnerability based on specific risk factors and individual characteristics, such as health status and social determinants. In contrast, subjective factors encompass openness, resources, sources of personal growth, development, and empathy. They acknowledge that a potential threat may not necessarily be perceived as such by the individual. Likewise, risk factors do not inherently exacerbate vulnerability; rather, their impact is contingent upon the individual’s appraisal of manageability, coping capacity, and available support mechanisms.

**Fig. 4:**
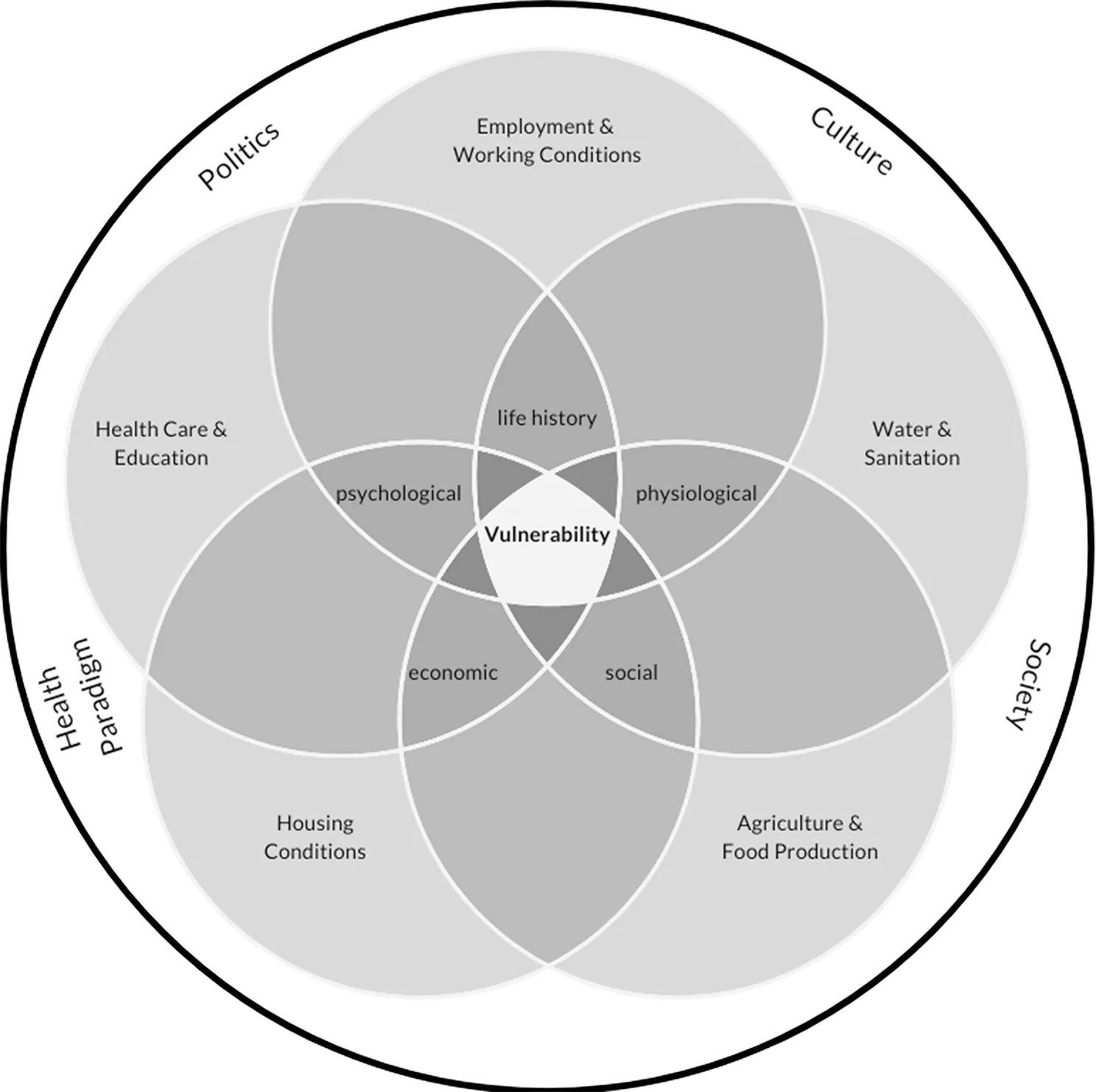
Relations of Vulnerability in Nursing Science

The results indicate that vulnerability in nursing science reflects a complex condition concerning its multidimensionality, as shown in Fig. 4.

Figure 4 illustrates how vulnerability requires a multidimensional understanding in nursing. The elements and dimensions of vulnerability overlap and influence each other. The core elements of Fig. 4 are briefly explained below.

The *individual dimension* in the center links to psychological, physiological, social, economic, and life-historical characteristics, often used to group people with similar attributes or variables to measure vulnerability by estimating risk or susceptibility and evaluating undesirable outcomes.

The *social dimension* refers to the social determinants of health: Water and sanitation, agriculture and food production, housing conditions, health care and education, and employment and working conditions. Potential economic crises are related to the latter. These environmental and socioeconomic factors are antecedents by shaping individual life history, experiences, and the ability to thrive and develop across life settings.

At the periphery, *institutional and programmatic state-level factors*, including politics, culture, and society, define the social dimension by shaping moral values, tolerance, freedom of expression, legislative frameworks, economic trajectories, and broader social development. The health paradigm acts as a distinct programmatic factor encompassing the sector’s strategic objectives; positions within capitalist versus socialist paradigms, curative versus preventive approaches, and biomedical versus health promotional models directly and indirectly modulate health care. These considerations determine the scope, design, and implementation of population-level interventions aimed at optimizing health and social outcomes.

### Regional use of perspectives on vulnerability

The review identified regional trends regarding perspectives on vulnerability. Studies from Brazil focus on a (3) public health-oriented perspective, assessing and managing vulnerability through socioeconomic, political, and cultural views. In these studies, health outcomes are described as primarily influenced by social determinants, such as poverty and adverse employment conditions, rather than by individual responsibility alone [31, 37, 39, 43]. North American and European studies tend to focus on (2) biomedical aspects and address vulnerable groups at higher risk. Researchers analyze risk factors to address their vulnerability in terms of susceptibility through tailored measures and special (institutional) services. Alongside these services, European contributions tend to approach to research ethics and subjective perspectives on vulnerability as a (1) basic human condition [9, 15, 33, 35, 61].

### Definition of vulnerability in nursing science

Building on the results, we propose the following preliminary definition of vulnerability within nursing science:

Vulnerability in nursing science represents an inherent human condition. Its manifestation is shaped by individual, programmatic, environmental, institutional, and sociocultural dimensions. Life circumstances—reflected by the dynamic interplay of subjective and objective factors—directly or indirectly modulate this vulnerability for better or for worse.

## Discussion

The principle-based concept analysis yields a definition that frames vulnerability in nursing science as a complex, multidimensional condition. In the following, we will discuss how this proposed definition aligns with the three identified perspectives: as (1) human condition, (2) individual, biomedical and risk-oriented, and (3) public health oriented, and what implications arise for the discipline. Figures 3 and 4 demonstrate that vulnerability is complex and multidimensional due to its relational, environmental, and situational influencing properties. Figure 4 illustrates the interfaces between the levels of programmatic, social determinants of health, and individual factors. These interfaces represent the dynamical character inherent in the concept of vulnerability: The mechanisms through which social determinants of health influence individuals in combination with their personal background, socialization, available resources, coping strategies, and life experiences remain empirically complex. While some individuals demonstrate resilience in challenging conditions and situations, others exhibit heightened susceptibility. Theoretically, constructs such as health literacy and individual sense of coherence provide valuable explanatory frameworks in this regard [62]. The influence of programmatic and institutional factors on social determinants of health adds to the complexity. Given that, the literature review suggests that individual agency plays a limited role in vulnerability patterns, with a strong emphasis placed on structural determinants that are difficult to modify directly by the individuals themselves. Consequently, addressing vulnerability becomes a central task for nursing science and practice. Drawing on salutogenic concepts of health [63], the discipline is called upon to operate across multiple levels: applying promotion theories at the individual level, actively engaging with social determinants at the structural level, and guiding policies and institutions at the programmatic level to effectively mitigate vulnerability.

### Discussion of the concept’s content and relevance for nursing

In nursing science, the individualistic perspective is mainly prevalent [12]. We propose interpreting this finding as a reflection of how the discipline is embedded in neoliberal attitudes and discourses, which tend to frame issues of health and care primarily as matters of individual responsibility rather than structural determinants [46, 64, 65]. Therefore, the concept of vulnerability evokes a disciplinary discussion about overarching philosophical questions regarding system design and nurses’ roles and responsibilities in capitalist or socialist healthcare systems. There is already literature about the extent to which nursing is conceived as a total institution, debating how nursing contributes to supporting capitalist healthcare systems and prioritizing the interests of the system over the needs of those being cared for. Other examples address nursing’s responsibility to ensure access to care, as related to philosophical ideas of kinship, solidarity, resistance, and community [66–68]. Thus, a nuanced conceptualization of vulnerability may serve as a critical lens for the discipline. It offers wider implications for promoting human flourishing and provides a way to question and rethink nursing’s approach to people across all settings. Applying this concept is not only important for individual care but is also crucial for fostering a healthy, functioning, and critical society.

Some of the literature reviewed emphasizes that risk and vulnerability are distinct concepts [51, 69]; however, the terms are often used synonymously [12, 56]: Empirical studies often use vulnerability as a categorical label, even when the construct is more precisely susceptibility, frailty, or risk. Recent work on conceptualizing vulnerability [57, 70, 71] focus on a dynamic relationship between care competencies and care needs, proposing that growing imbalances can lead to frailty. This approach provides an operational framework for assessing and measuring vulnerability in nursing practice. In contrast, the concept developed in this analysis adopts a broader multidimensional perspective that incorporates social determinants of health and institutional factors, alongside individual characteristics. Figure 4’s interfaces highlight the as yet unquantified interactions between the several dimensions that shape people during their lifetime. Coping capacity varies across individuals and in different stages of life, encompassing heterogeneous adaptation to adverse contexts. The included studies do not explicitly articulate links between health literacy, sense of coherence, and vulnerability. However, established connections among the components of sense of coherence and health literacy [72, 73] indicate a plausible pathway: Investing in health promotion and health literacy is the key to reducing individual vulnerability. Figure 4 is a first exploratory approach to conceptualizing and plotting the multidimensional influences within vulnerability.

### Discussion of the concept’s maturity

The analysis of the included studies reveals three principal perspectives on vulnerability, each demonstrating maturity in its respective domain of application. Figure 4’s conceptual framework offers a model that methodologically integrates diverse viewpoints and providing a theoretical basis for future empirical work.

Pragmatically, the concept is not yet operationalized well. The three perspectives on vulnerability are referred to within different methodological approaches which partially result in evaluated assessment tools [52]. However, the qualitative, self-assessed approach is often not addressed [38] and specifically, the possibility of an empirical access approach to perceived embodied vulnerability in nursing remains to be explored. Further, there is currently no assessment tool that incorporates the integrative concept in Fig. 4.

Linguistically, use of vulnerability is inconsistent across the three perspectives. Theoretically, it should be distinguished from closely related concepts such as susceptibility, frailty, and risk, yet this demarcation is often blurred. This concept analysis therefore acknowledges the research gaps between vulnerability, risk, frailty, and susceptibility. For nursing, a theoretical distinction of the concepts is needed and an examination of their logical relationships in order to use them in a reflective and substantiated manner.

Logically, the observed terminological inconsistency constrains a claim of full maturity. A mature construct displays consistent usage, clear discrimination from closely related concepts, and robust operationalization (e.g., agreed definitions, measures, and applications across settings). Furthermore, addressing these interconnected aspects requires a future operationalization and assessment framework for vulnerability that integrates individuals’ life circumstances, political contexts, and social determinants of health, rather than focusing solely on biomedical or individualistic factors [50, 60]. On this basis, the present concept analysis contributes to the conceptualization of vulnerability within nursing science while supporting its classification as a partially mature concept.

### Discussion of methodology

While concepts are often portrayed as foundational “building blocks” of nursing theory [19, 74], this view has been criticized, and some authors have rejected it entirely [75, 76]. Nevertheless, concepts can enhance the theoretical understanding of a specific topic within a particular context. Especially the principle-based concept analysis offers a transparent and structured procedure for synthesizing from concepts from the scientific evidence base. By evaluating a concept across four dimensions, it enables explicit assessment and identifies priorities for targeted further development. Methodologically, the approach is designed to synthesize knowledge across disciplinary domains [16].

The discussion about evaluating the maturity of concepts is more than 25 years old. At that time, the debate about feminist epistemology also took place, addressing situated knowledge and standpoint epistemology in science [77, 78]. In terms of classifying concepts as either mature or immature, feminist epistemology might contend that mature concepts strive to generate objective, unbiased theoretical and empirical knowledge—thereby overlooking standpoint perspectives and power relations in the process of scientific inquiry. This contention is understandable concerning a traditional way of understanding concepts as fixed building blocks of theory; however, our reasoning was quite the opposite when we chose the method of principle-based concept analysis: Our analysis is a first step in identifying and presenting different perspectives on the concept of vulnerability. This manuscript starts with reflections in nursing science due to the affiliations of the authors. Therefore, perspectives from nursing ethics or caring science are only marginally represented at this point in time and need to be integrated and discussed in a further step. Then again, discourses, power relations, and standpoint perspectives must be taken into account.

Methodically, applying the principles enables an equal and non-hierarchical analysis of all perspectives, thereby developing an integrative understanding of the term “vulnerability”. This concept analysis embeds and transforms the dominant North American and European biomedical perspective within an understanding of social deterministic and anthropological perspectives. This reduces the naturalist-individualistic notions of the biomedical perspective and—rooted in anthropological and philosophical approaches—it reflects nursing’s universalistic approach [10], providing support for individuals facing health-impacting crises.

After clarifying the concept in the field of nursing and care, it should be examined from a multidisciplinary perspective to evaluate the contributions of each relevant discipline and distinguish discipline-specific meanings from those in adjacent fields.

### Limitations

First, the analysis focused on the discipline of nursing science. Consequently, perspectives from adjacent fields—specifically sociology, philosophy, and ethics—were excluded unless explicitly discussed within the nursing context. While nursing literature frequently references these traditions, it often prioritizes disciplinary utility over deep philosophical exploration. Therefore, the resulting definition may not fully capture the anthropological or ontological depth of vulnerability as understood in philosophy or the humanities. Furthermore, the search was restricted to indexed sources in MEDLINE (via PubMed), CINAHL, and Livivo. Although citation searching was performed to broaden the retrieval, potentially relevant international gray literature may have been missed. Furthermore, other combinations of the Boolean Operators may have led to additional papers discussing the conceptual boundaries between vulnerability, susceptibility, risk, and frailty. However, this finding was mentioned in several studies included in our literature review.

Second, regarding the methodology, the principle-based concept analysis relies on the four criteria established by Penrod and Hupcey [16]: the epistemological, pragmatic, linguistic, and logical principle. While this framework is suitable for assessing a concept’s maturity and utility within the discipline, it does not address additional perspectives, such as ontology, axiology, values, or ethics for philosophical inquiry. Therefore, the analysis presents vulnerability predominantly through a pragmatic-epistemic lens. Ontological questions regarding the nature of being human and ethical considerations remain largely unaddressed within this specific analytical framework. The method was deliberately chosen to clarify the current use and definition of vulnerability in nursing science; thus, integrating explicit ontological or ethical dimensions is identified as a task for subsequent research.

Third, the theoretical model presented in Fig. 4 is derived from an interpretive synthesis of the reviewed literature. It currently lacks empirical validation confirming the proposed relationships. However, it is intended to be refined and validated upon the integration of perspectives from adjacent disciplines in future research projects.

## Conclusions

This concept analysis shows that vulnerability is a multidimensional construct structured by a variety of factors and institutions across individual, interpersonal, organizational, and socio-political levels. Social determinants of health are produced through political, societal, and cultural processes, as well as by the prevailing health paradigm. They shape life conditions and opportunities, including sense of coherence and health literacy. Under these structural arrangements, individuals have limited scope for health-related decision-making. It is problematic to expect that individuals and nurses are able to maintain and promote health within systems that neglect structural barriers to resources and access to health care. In the context of nursing, it is imperative for the discipline to critically assess the extent to which social and healthcare systems are designed to recognize, accommodate, and address vulnerability in its multifaceted forms. A foundational step in advancing this discourse involves systematically examining the influence of vulnerability on health and social outcomes, thereby informing more equitable and responsive healthcare models.

## Supplementary Information

Below is the link to the electronic supplementary material.

Supplementary Material 1

Supplementary Material 2

## Data Availability

No datasets were generated or analysed during the current study.

## Footnotes

1 Since it is not known whether the people affected would also describe themselves as vulnerable, quotes or the term “so-called vulnerable groups of people” are used here.

2 The PRISMA-ScR Guideline [20] does not provide a specific version of the flow chart but refers to the actual version of the PRISMA statement flow chart [21], which is referred to here accordingly.

3 Further sources [19, 28] provide an overview of different approaches, evolutions, and critical appraisals of concept development and concept analysis.

4 Programmatic factors include policy, politics, and the provision of structural services that enable health and nursing care to be provided at the individual and societal levels. At the macro level, this involves political action and legislation. At the institutional level, it encompasses leadership, management concepts, and business decisions regarding the type of care provided.

## Declarations

### Data Sharing

Data sharing is not applicable to this article as no datasets were generated or analysed during the current study.

## References

[1] Oxford English Dictionary. Oxford University Press; 2023.

[2] de Macedo Couto R, Santos GonçalvesD, Rodrigues Sanine D, Pires Dos Santos P, Bach Kmetiuk A, Welker Biondo L, Crispim Boing A. Mapping Vulnerability for Increased COVID-19 Susceptibility and Worse Outcomes: a Scoping Review. Front Public Health. 2024;12:1432370. 10.3389/fpubh.2024.1432370. PMC11499102 39450391

[3] Scully JL. Disability and Vulnerability. In: Mackenzie C, Rogers W, Dodds S, editors. Vulnerability: New Essays in Ethics and Feminist Philosophy. New York: Oxford University Press; 2013. pp. 204–21. 10.1093/acprof:oso/9780199316649.003.0009.

[4] Tiliouine H, Benatuil D, Lau MKW. Handbook of Children’s Risk, Vulnerability and Quality of Life: An Introduction. In: Tiliouine H, Benatuil D, Lau MKW, editors. Handbook of Children’s Risk, Vulnerability and Quality of Life: Global Perspectives. Cham: Springer International Publishing; 2022. pp. 1–10.

[5] Nature Progress Report. How Epidemiology has Shaped the COVID Pandemic. Nature. 2021;589:491–2. 10.1038/d41586-021-00183-z. 33505041

[6] Pedroso MLR, Da Motta MGC. Comprehension of Social and Economic Vulnerability in the Scenario of Pediatrics Nursing Care. Revista Gaúcha de Enfermagem. 2010;31:218–24. 10.1590/s1983-14472010000200003. 21500499

[7] Hino P, Takahashi RF, Nichiata LYI, Apostólico MR, Taminato M, Fernandes H. Interfaces of Vulnerability Dimensions in Violence Against Children. Revista Brasileira de Enfermagem. 2019;72:343–7. 10.1590/0034-7167-2018-0463. 31851272

[8] Scholz S, Zerth J, editors. Versorgung gestalten in vulnerablen Lebenslagen. Stuttgart: Verlag W. Kohlhammer; 2024.

[9] Schrems BM. Informed Consent, Vulnerability and the Risks of Group-Specific Attribution. Nurs Ethics. 2014;21:829–43. 10.1177/0969733013518448. 24493709

[10] International Council of Nurses (ICN). Renewing the definitions of ‘nursing’ and ‘a nurse’. Geneva; 2025.

[11] World Health Organization. Primary health care measurement framework and indicators: monitoring health systems through a primary health care lens. Geneva; 2021.

[12] Nichiata LYI, Bertolozzi MR, Takahashi RF, Fracolli LA. The Use of the Vulnerability Concept in the Nursing Area. Revista Latino-Americana Enfermagem. 2008;16:923–8. 10.1590/s0104-11692008000500020. 19061032

[13] Butler J. Precarious Life: The Powers of Mourning and Violence. London: Verso; 2004.

[14] Huth M, Thonhauser G, Introduction. Philos Today. 2020;64:537–55. 10.5840/philtoday2020643346.

[15] Heaslip V. Understanding Vulnerability. In: Heaslip V, Ryden J, editors. Understanding vulnerability: a nursing and healthcare approach. Wiley-Blackwell; 2013. pp. 6–27.

[16] Penrod J, Hupcey JE. Enhancing methodological clarity: principle-based concept analysis. J Adv Nurs. 2005;50:403–9. 10.1111/j.1365-2648.2005.03405.x. 15842447

[17] Morse JM. Exploring the Theoretical Basis of Nursing using Advanced Techniques of Concept Analysis. Adv Nurs Sci. 1995;17:31–46. 10.1097/00012272-199503000-00005. 7778889

[18] Morse JM, Mitcham C, Hupcey JE, Tasón MC. Criteria for Concept Evaluation. J Adv Nurs. 1996;24:385–90. 10.1046/j.1365-2648.1996.18022.x. 8858445

[19] Morse JM, Hupcey JE, Mitcham C, Lenz ER. Concept Analysis in Nursing Research: A Critical Appraisal. Sch Inq Nurs Pract. 1996;10:253–77. 10.1891/0889-7182.10.3.253. 9009821

[20] Tricco AC, Lillie E, Zarin W, O’Brien KK, Colquhoun H, Levac D, et al. PRISMA Extension for Scoping Reviews (PRISMA-ScR): Checklist and Explanation. Annuals Intern Med. 2018;169:467–73. 10.7326/M18-0850. 30178033

[21] Page MJ, McKenzie JE, Bossuyt PM, Boutron I, Hoffmann TC, Mulrow CD, et al. The PRISMA 2020 Statement: an Updated Guideline for Reporting Systematic Reviews. BMJ. 2021;372:n71. 10.1136/bmj.n71. PMC8005924 33782057

[22] Haddaway NR, Grainger MJ, Gray CT. Citationchaser: an R Package and shiny app for forward and backward citations chasing in academic searching. Zenodo; 2021.

[23] Hirt J, Nordhausen T, Fuerst T, Ewald H, Appenzeller-Herzog C. Guidance on Terminology, Application, and Reporting of Citation Searching: the TARCiS statement. BMJ. 2024;385:e078384. 10.1136/bmj-2023-078384. 38724089

[24] Ouzzani M, Hammady H, Fedorowicz Z, Elmagarmid A. Rayyan-a Web and Mobile app for Systematic Reviews. Syst Reviews. 2016;5:210. 10.1186/s13643-016-0384-4. PMC5139140 27919275

[25] VERBI GmbH Berlin. MAXQDA Plus; 2024.

[26] Walker L, Avant KC. Strategies for Theory Construction in Nursing: Pearson New International Edition. 5th ed. Harlow: Pearson Education Limited; 2013.

[27] Wilson J. Thinking with concepts. Cambridge University Press; 2012.

[28] Paley J. Reading Concept Analysis: Why Draper has a point. Nurs Philos. 2019;20:e12252. 10.1111/nup.12252. 31136083

[29] Hupcey JE, Penrod J. Concept analysis: Examining the State of the Science. Res Theory Nurs Pract. 2005;19:197–208. 16025698

[30] Haddaway NR, Page MJ, Pritchard CC, McGuinness LA. PRISMA2020: An R package and Shiny app for producing PRISMA 2020-compliant flow diagrams, with interactivity for optimised digital transparency and Open Synthesis. Campbell Syst Reviews. 2022;18:e1230. 10.1002/cl2.1230. PMC8958186 36911350

[31] Cestari VRF, Moreira TMM, Pessoa RS, Da Silva MRF, Torres RAM. The essence of care in health vulnerability: a heideggerian construction. Revista Brasileira de Enfermagem. 2017;70:1112–6. 10.1590/0034-7167-2016-0570. 28977242

[32] Sarvimäki A, Stenbock-Hult B. The Meaning of Vulnerability to Older Persons. Nurs Ethics. 2014;23:372–83. 10.1177/0969733014564908. 25552588

[33] Sellman D. Towards an Understanding of Nursing as a Response to Human Vulnerability. Nurs Philos. 2005;6:2–10. 10.1111/j.1466-769X.2004.00202.x 15659085

[34] Malone RE. Dimensions of Vulnerability in Emergency Nurses’ Narratives. Adv Nurs Sci. 2000;23:1–11. 10.1097/00012272-200009000-00005. 10970035

[35] Angel S, Vatne S. Vulnerability in Patients and Nurses and the Mutual Vulnerability in the Patient-Nurse Relationship. J Clin Nurs. 2017;26:1428–37. 10.1111/jocn.13583. 27626897

[36] Spiers J. New Perspectives on Vulnerability Using Emic and Etic Approaches. J Adv Nurs. 2000;31:715–21. 10.1046/j.1365-2648.2000.01328.x. 10718892

[37] Silva DI, Peres AM, Gonçalves Wolff LD, de Azevedo Mazza V. Contributions of the Concept of Vulnerability to Professional Nursing Practice: Integrated Review. Revista de Pesquisa Cuidado é Fundamental. 2014;6:848–55. 10.9789/2175-5361.2014v6n2p848.

[38] de Santis J. Exploring the Concepts of Vulnerability and Resilience in the Context of HIV Infection. Res Theory Nurs Pract. 2008;22:273–87. 10.1891/0889-7182.22.4.273. 19093664

[39] Gabrício Marçola A, Cabett Cipolli G, Casadei Donatelli D, Carneiro Júnior N. Barbosa do Nascimentoa V. A Look at Vulnerability in the Older Population in Health Sciences Studies: A Systematic Review. Geriatr Gerontol Aging. 2023;17:e0230021. 10.53886/gga.e0230021.

[40] Lima Costa LE, Da Souza Silva R, Dias Quirino M. Differents Ways from Existence: Concept of Vulnerability in People living with Deficiency. Revista de Enfermagem UFPE Line. 2010;4:412–16.

[41] de Sousa PKR, Miranda KCL, Franco AC. Vulnerability: Concept Analysis in the Clinical Practice of Nurses from Outpatient HIV/AIDS. Revista Brasileira de Enfermagem. 2011;64:381–4. 10.1590/s0034-71672011000200026. 21755227

[42] Zarth MD, Fernández PA, Baggio MA, Zilly A, Gamarra CJ, Silva. Rosane Meire Munhak da. Cross-Cultural Nursing Care for Immigrant Women During Pregnancy and Childbirth: Experiences and Vulnerabilities. Revista Gaúcha de Enfermagem. 2024;45:e20230161. 10.1590/1983-1447.2024.20230161.en. 38922234

[43] Pedroso MLR, Da Motta MGC. Socio-Economic Vulnerability and Pediatric Nursing Care Routine: Nurses Report. Escola Anna Nery. 2010;14:293–300.

[44] Ayres JoséRdeC, Mesquita, Calazans GJ, Saletti Filho HC, Franca Junior I, Risco. Vulnerabilidade e Práticas de Prevenção e Promoção da Saúde. In: Campos, Gastão Wagner de Sousa, Minayo, Maria Cecília de Souza, Akerman M, Drumond Júnior M, Carvalho YM de, editors. Tratado de Saúde Coletiva. São Paulo: Hucitec; 2006. pp. 375–417.

[45] de Lima Trindade L, Ferraz L, Ferraboli SF, Rubini B, Trevisan Saldanha C, Bordignon M, Vendruscolo C. Vocational Training in Directing Assistance to Vulnerable Groups in Primary Care. Revista de Enfermagem da Universidade Fed de Santa Maria. 2015;5:368–78. 10.5902/2179769213738.

[46] Tomm-Bonde L. The Naïve Nurse: Revisiting Vulnerability for Nursing. BMC Nurs. 2012;11:5. 10.1186/1472-6955-11-5. PMC3531306 22520841

[47] Carel H. A Reply to ‘Towards an Understanding of Nursing as a Response to Human Vulnerability’ by Derek Sellman: Vulnerability and Illness. Nurs Philos. 2009;10:214–9. 10.1111/j.1466-769X.2009.00401.x. 19527442

[48] Kipnis K. Vulnerability in Research Subjects. In: Weisstub DN, Thomasma DC, editors. The Variables of Moral Capacity. Dordrecht: Springer Netherlands; 2004. pp. 217–31. 10.1007/978-1-4020-2552-5_16.

[49] Heaslip V, Hean S, Parker J. The Etemic Model of Gypsy Roma Traveller Community Vulnerability: is it Time to Rethink our Understanding of Vulnerability? J Clin Nurs. 2016;27:3426–35. 10.1111/jocn.13499. 27505706

[50] Nyamathi A, Koniak-Griffin D, Greengold BA. Development of Nursing Theory and Science in Vulnerable Populations Research. Annu Rev Nurs Res. 2007;25:3–25. 10.1891/0739-6686.25.1.3. 17958287

[51] Carvalho Barra DC, Marcellino de Melo Lanzoni, Gabriela, Alves Maliska IC, Sebold LF. Hörner Schlindwein Meirelles B. Human Living process and nursing from the vulnerability perspective. Acta Paulista de Enfermagem. 2010;23:831–6.

[52] Levasseur M, Lussier-Therrien M, Biron ML, Dubois M-F, Boissy P, Naud D, et al. Scoping Study of Definitions and Instruments Measuring Vulnerability in Older Adults. J Am Geriatr Soc. 2022;70:269–80. 10.1111/jgs.17451. 34669967

[53] Bandura A. Social Foundations of Thought and Action: A Social Cognitive Theory. Englewood Cliffs, NJ: Prentice-Hall; 1986.

[54] Williams DM. Vulnerable Families: a Study of Health Visitors’ Prioritization of their Work. J Nurs Adm Manag. 1997;5:19–24. 10.1046/j.1365-2834.1997.02413.x. 9146200

[55] MacLellan J. Vulnerability in Birth: A Negative Capability. J Clin Nurs. 2020;29:3565–74. 10.1111/jocn.15205. 32045047

[56] Dorsen C. Vulnerability in Homeless Adolescents: Concept Analysis. J Adv Nurs. 2010;66:2819–27. 10.1111/j.1365-2648.2010.05375.x. 21073499

[57] Fernández Batalla M, Monsalvo San Macario E, González Aguña A, Santamaria García JM. Diseño de un Método de Análisis para el Cálculo de la Vulnerabilidad como Predictor de la Fragilidad en Salud. Revista de Enfermería. 2018;12.

[58] Brocklehurst H, Laurenson M. A Concept Analysis Examining the Vulnerability of Older People. Br J Nurs. 2008;17:1354–7. 10.12968/bjon.2008.17.21.31738. 19060820

[59] Heaslip V, Ryden J, editors. Understanding vulnerability: a nursing and healthcare approach. Wiley-Blackwell; 2013.

[60] Heaslip V, Hean S, Parker J. Lived Experience of Vulnerability from a Gypsy Roma Traveller Perspective. J Clin Nurs. 2016;25:1987–98. 10.1111/jocn.13223. 27002227

[61] Eriksen KÅ, Sundfør B, Karlsson B, Råholm M-B, Arman M. Recognition as a Valued Human Being: Perspectives of Mental Health Service Users. Nurs Ethics. 2012;19:357–68. 10.1177/0969733011423293. 22581505

[62] Moksnes UK. Health promotion in health care – vital theories and research: sense of coherence. Cham; 2021.

[63] Dahlgren G, Whitehead M. Policies and strategies to promote social equity in health. background document to WHO-strategy paper for Europe; 2007.

[64] Traynor M. The Promotion of Resilience in Nursing. In: Lipscomb M, editor. Routledge Handbook of Philosophy And Nursing. S.l.: Routledge; 2023. pp. 239–49. 10.4324/9781003427407-28.

[65] Navarro V. Neoliberalism as a Class Ideology; or, the Political Causes of the Groth of Inequalities. Int J Health Serv. 2007;37:47–62. 10.2190/AP65-X154-4513-R520 17436985

[66] Dillard-Wright J, Walsh JH, Brown BB. We Have Never Been Nurses: Nursing in the Anthropocene, Undoing the Capitalocene. Adv Nurs Sci. 2020;43:132–46. 10.1097/ANS.0000000000000313. 32345801

[67] Dillard-Wright J. Another nursing is possible: Ethics, Political Economies, and Possibility in an Uncertain World. Nurs Philos. 2024;25:e12484. 10.1111/nup.12484. 38739847

[68] Smith KM, Foth T. Tomorrow is Cancelled: Rethinking Nursing Resistance as Insurrection. Aporia. 2021;13:15–25.

[69] Mattheus DJ. Vulnerability Related to Oral Health in Early Childhood: a Concept Analysis. J Adv Nurs. 2010;66:2116–25. 10.1111/j.1365-2648.2010.05372.x. 20626483

[70] Fernández Batalla M. Valoración de la Vulnerabilidad a Través de las Competencias y Necesidades de Cuidado. Revista de Enfermería. 2019;13:1–10.

[71] Fernández Batalla M, Monsalvo San Macario E, González Aguña A, Herrero Jaén S, Gonzalo de Diego B, Manrique Anaya Y, et al. Validation and Reliability of the Care Vulnerability Index: A Study by Interrater Agreement and test-retest Method. Nurs Open. 2022;9:1766–73. 10.1002/nop2.1203. PMC8994951 35261198

[72] Hallberg LR, Carlsson SG. Psychosocial Vulnerability and Maintaining Forces related to Fibromyalgia. In-depth Interviews with twenty-two female Patients. Scand J Caring Sci. 1998;12:95–103. 9801630

[73] Sørensen K, van den Broucke S, Fullam J, Doyle G, Pelikan J, Slonska Z, Brand H. Health Literacy and Public Health: a Systematic Review and Integration of Definitions and Models. BMC Public Health. 2012;12:80–93. 10.1186/1471-2458-12-80. PMC3292515 22276600

[74] Hellman AN. The Concept Analysis: An Effective and Important Starting Point in Nursing Research. J Radiol Nurs. 2024;43:11–4. 10.1016/j.jradnu.2023.12.001.

[75] Paley J. Concept Analysis. In: Lipscomb M, editor. Routledge Handbook of Philosophy And Nursing. S.l.: Routledge; 2023. pp. 578–91.

[76] Bergdahl E, Berterö CM. Concept Analysis and the Building Blocks of Theory: Misconceptions Regarding Theory Development. J Adv Nurs. 2016;72:2558–66. 10.1111/jan.13002. 27218439

[77] Haraway D. Situated Knowledges: The Science Question in Feminism and the Privilege of Partial Perspective. Feminist Stud. 1988;14:575–99. 10.2307/3178066.

[78] Harding S. Rethinking Standpoint Epistemology: What is Strong Objectivity? Centennial Rev. 1992;36:437–70.

