# Supplementary material for: Vulnerability in nursing science: a principle-based concept analysis
Source: BMC Nurs. 2026 Oct 16;25:989. doi: 10.1186/s12912-026-05523-w (PMC13675958; doi:10.1186/s12912-026-05523-w)
Supplement: Supplementary file 2 [file 12912_2026_5523_MOESM2_ESM.docx]

**Supplemental Material**

# A: Database search (Jan 20, 2025)

MEDLINE (PubMed)

| # | Entry | Hits |
| --- | --- | --- |
| 1 | “Vulnerability“[all fields] | 82.317 |
| 2 | “Nursing“[MeSH] | 268.220 |
| 3 | “Concept”[all fields] OR “phenomenon”[all fields] OR “understanding”[all fields] | 1.940.674 |
| 4 | #1 AND #2 AND #3 | 171 |

CINAHL

| # | Entry | Hits |
| --- | --- | --- |
| 1 | TX “Vulnerability“ | 27.024 |
| 2 | MM “Nursing Practice“ | 26.934 |
| 3 | TX “Concept” OR TX “phenomenon” OR TX “understanding” | 397.343 |
| 4 | #1 AND #2 AND #3 | 39 |

LIVIVO

| # | Entry | Hits |
| --- | --- | --- |
| 1 | OS=“Vulnerability“ | 137.116 |
| 2 | KW=“Nursing“ | 670.942 |
| 3 | OS=“Concept” OR OS=“phenomenon” OR OS=“understanding” | 2.876.214 |
| 4 | #1 AND #2 AND #3 | 366 |

# B: Data Extraction Sheet, used in MaxQDA 24

**Structural Details**

- Author(s)
- Title
- Publication Year
- Origin Country of the Corresponding Author
- Study Design and Methods

**Contents**

- Epistemological Principle
- Pragmatic Principle
- Linguistic Principle
- Logical Principle
- Antecedents
- Attributes
- Consequences
